# Propofol Requirement for Induction of Unconsciousness Is Reduced in Patients with Parkinson's Disease: A Case Control Study

**DOI:** 10.1155/2015/953729

**Published:** 2015-10-01

**Authors:** Xiao-ping Xu, Xi-ya Yu, Xi Wu, Xiao-wu Hu, Jian-chun Chen, Jin-bao Li, Jia-feng Wang, Xiao-ming Deng

**Affiliations:** ^1^Department of Anesthesiology and Intensive Care Medicine, The Second Military Medical University, 168 Changhai Road, Shanghai 200433, China; ^2^Department of Anesthesiology, Affiliated Union Hospital of Fujian Medical University, 29 Xinquan Road, Fuzhou, Fujian 350001, China; ^3^Department of Neurosurgery, Changhai Hospital, The Second Military Medical University, 168 Changhai Road, Shanghai 200433, China

## Abstract

Parkinson's disease (PD) is the second most prevalent neurodegenerative disease, but whether the neurodegenerative process influences the pharmacodynamics of propofol remains unclear. We aimed to evaluate the effect of PD on pharmacodynamics of propofol. A total of 31 PD patients undergoing surgical treatment (PD group) and 31 pair-controlled non-PD patients undergoing intracranial surgery (NPD group) were recruited to investigate the propofol requirement for unconsciousness induction. Unconsciousness was induced in all patients with target-controlled infusion of propofol. The propofol concentration at which unconsciousness was induced was compared between the two groups. EC_50_ and EC_95_ were calculated as well. Demographic data, bispectral index, and hemodynamic values were comparable between PD and NPD groups. The mean target concentration of propofol when unconsciousness was achieved was 2.32 ± 0.38 *μ*g/mL in PD group, which was significantly lower than that in NPD group (2.90 ± 0.35 *μ*g/mL). The EC_50_ was 2.05 *μ*g/mL (95% CI: 1.85–2.19 *μ*g/mL) in PD group, much lower than the 2.72 *μ*g/mL (95% CI: 2.53–2.88 *μ*g/mL) in NPD group. In conclusion, the effective propofol concentration needed for induction of unconsciousness in 50% of patients is reduced in PD patients. (This trial is registered with NCT01998204.)

## 1. Introduction

Parkinson's disease (PD) is the second most prevalent neurodegenerative disease in the world, with an increasing incidence among the elderly [1]. It is reported that the incidence is about 0.3% in the general population, but as high as 3% in patients over 65 years [2]. Anesthesia in PD patients has been considered a challenge because of disability of the patients and interactive reaction between anesthetics and anti-PD medications or PD symptoms [3, 4].

Deep brain stimulator (DBS) was first introduced to treat PD in 1987 [5, 6], sending electrical impulses to thalamus, subthalamic nucleus, and globus pallidus. Anesthesia concerns have been focused on the anesthesia type and withdrawal of anti-PD medications [7–9], but there is little information about the pharmacodynamic changes of anesthetic agents in PD patients undergoing DBS implantation because of the degenerate brain. We speculated that the imbalance of neurotransmitter might change the amount of anesthetics that patients required for anesthesia. The aim of the present study was to determine whether the requirement for propofol to induce unconsciousness was reduced in patients with Parkinson's disease.

## 2. Materials and Methods

### 2.1. Study Design

This prospective case control study was performed in our hospital from January 2012 to June 2013 upon the approval from the Ethics Committee of Biomedicine Research of The Second Military Medical University (Shanghai, China). Informed consent was obtained from all patients or their surrogates before recruitment. The trial protocol was registered as NCT01998204 in clinicaltrial.gov.

### 2.2. Subjects

A total of 31 adult PD patients undergoing DBS implantation and pulse generator placement were recruited in the Parkinson's disease group (PD group). The exclusion criteria included ASA score higher than class III, predicted difficult airway, hearing impairment, inability to follow instructions, alcohol or drug abusers, and patients who refused to provide informed consent.

Additional 31 patients undergoing intracranial surgery for tumors were assigned as non-PD (NPD) group based on a 1 : 1 pairing principle. Subjects in NPD group should be of the same sex and similar age (±3 years) to the counterparts in PD group.

### 2.3. Trial Protocol

All patients fasted for 8 h before surgery and received no premedication. They were administered with 10 mL/kg Ringer's solution and monitored with noninvasive blood pressure (BP), heart rate (HR), pulse oximetry (SpO_2_), electrocardiography (ECG), and bispectral index (BIS). Oxygen was provided at a 6 L/min rate before propofol (AstraZeneca, Italy) administration through a target controlled infusion (TCI) pump (Fresenius, German) using the Marsh model. A Chinese study reported that when plasma concentration of propofol was set to 2.0 *μ*g/mL, a concentration of 1.9 *μ*g/mL would induce unconsciousness in the population [10]; therefore, the target effect-site concentration of propofol was started at 1.4 *μ*g/mL. If unconsciousness was not induced when the target concentration was stabilized for one min, the target concentration was added by 0.2 *μ*g/mL. Consciousness was assessed again 20 s after unconsciousness was achieved, based on the observer's assessment of alertness and sedation score (OAA/S). The target concentration of propofol at the time of achieving unconsciousness was considered as the dose of propofol required to induce unconsciousness for this patient. Unconsciousness was defined as an OAA/S score not higher than 1 [11, 12]. Assisted respiration was performed by the anesthetic machine if SpO_2_ was lower than 92%. The vasopressor agent or atropine was administered if hypotension or bradycardia occurred.

### 2.4. Outcome

The primary outcome is the target concentration of propofol when unconsciousness was induced. BIS and hemodynamic variables were recorded before and after propofol induction.

### 2.5. Power Estimation

According to a clinical trial in a Chinese population [10], the mean standard derivation (SD) of propofol to reach an OAA/S score of 1 was 0.3–0.4 *μ*g/mL. In order to detect a disparity of 0.3 *μ*g/mL, at least 28 patients in each group should be included for a power of 0.8 and *α* = 0.05.

### 2.6. Statistical Analysis

All statistical analyses were performed in SPSS 16.0. Continuous data were expressed as mean ± SD. Intergroup comparison was accomplished by paired *t*-test or chi-square analysis between the two groups. The effective propofol concentrations needed for induction of unconsciousness in 50% (EC_50_) and 95% (EC_95_) of patients were calculated by probit regression, and EC_50_ of different groups was compared using the relative median potency estimates. A *P* < 0.05 was considered statistically significant.

## 3. Results

**Figure 1:**
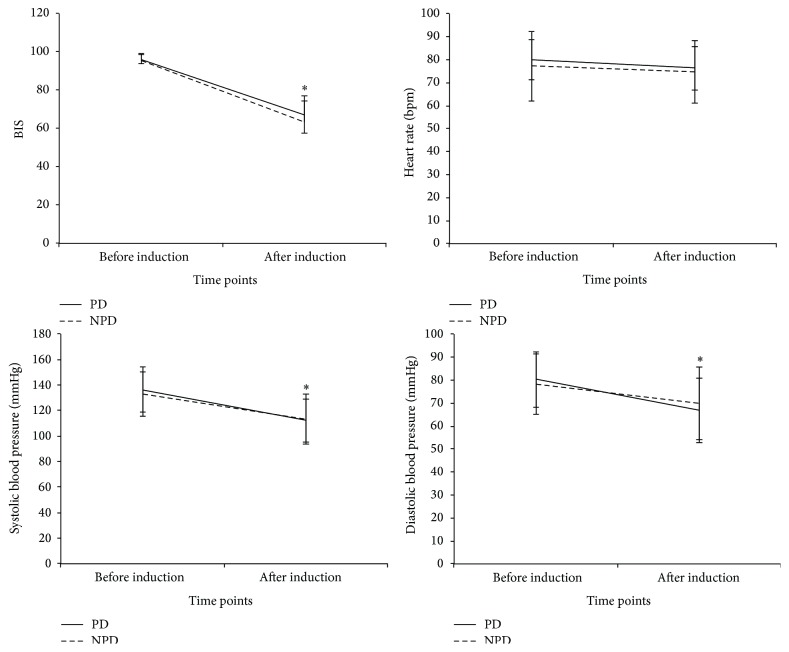
BIS, heart rate, systolic blood pressure, and diastolic blood pressure before and after propofol induction (*n* = 31 for both groups). PD: Parkinson's disease; NPD: non-Parkinson's disease; BIS: bispectral index. Results are given as mean (standard derivation). ^*∗*^ *P* < 0.05 compared with before induction in both groups.

All the 62 patients completed the study and none was excluded during the trial. General demographic data are shown in Table 1. Age, gender, body mass index (BMI), ASA classification, BIS, HR, and BP were comparable between PD and NPD groups. The medications that the PD patients were taking were listed in Table 2. As shown in Figure 1, propofol administration reduced BIS, systolic BP, and diastolic BP significantly (*P* < 0.05), but there was no significant difference between the 2 groups regarding BIS, HR, systolic BP, and diastolic BP.

**Figure 2:**
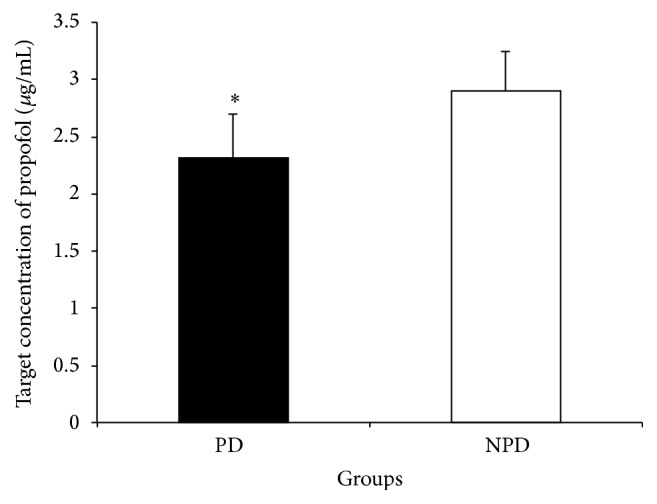
The target concentration of propofol when unconsciousness is induced by propofol. PD: Parkinson's disease; NPD, non-Parkinson's disease (*n* = 31 for both groups). Results are given as mean (standard derivation). ^*∗*^ *P* < 0.05 compared with NPD group.

The target concentration of propofol for induction of unconsciousness was 2.32 ± 0.38 *μ*g/mL in PD group, which was significantly lower than 2.90 ± 0.35 *μ*g/mL in NPD group (*P* < 0.05) (Figure 2). EC_50_ and EC_95_ were 2.05 *μ*g/mL (95% CI: 1.85–2.19 *μ*g/mL) and 2.91 (95% CI: 2.75–3.15 *μ*g/mL) in PD group and 2.72 *μ*g/mL (95% CI: 2.53–2.88 *μ*g/mL) and 3.59 *μ*g/mL (95% CI: 3.39–3.88 *μ*g/mL) in NPD group. Comparison of EC_50_ between the two groups showed that EC_50_ in PD group was significantly lower than that in NPD group, since the relative median potency estimate was 0.677 (95% confidential interval: 0.368, 1.156).

## 4. Discussions

Our study demonstrated that the propofol requirement for induction of unconsciousness was reduced in PD patients undergoing DBS implantation and pulse generator placement. The mean target concentration at the time of achieving unconsciousness and EC_50_ of propofol for unconsciousness induction were lower in PD patients than those in NPD patients.

Our data is important for clinical anesthesia, because the prevalence of PD is reported to be as high as 3% in patients older than 65 years [2], which raises concerns over the anesthesia management in PD patients. Unfortunately, most of these concerns were focused on the anesthetic techniques for DBS implantation or interaction between anesthetics and chronic medications or PD symptoms [3, 4]. To the best of our knowledge, this is the first work showing that the propofol requirement for unconsciousness induction was reduced in PD patients, which might change our anesthetic techniques among a proportion of patients older than 65 years. The conventional pharmacodynamic concept may lead to the relative overdose of propofol in this population and further result in compromise of cardiovascular function, delayed emergence, and postoperative delirium due to oversedation [13]. Deep anesthesia with a BIS lower than 20 has been demonstrated as an independent predictor for postoperative delirium. Moreover, BIS-guided anesthesia to reduce propofol delivery during anesthesia was believed to be protective against postoperative cognitive dysfunction [14]. Therefore, oversedation produced by the relatively lower requirement of propofol should be concerned in PD patients.

It remained unclear why PD patients required a lower dose of propofol than did NPD patients. The reason might include neurodegenerative changes during PD progression and anti-PD medications. The loss of dopaminergic neurons and reduced dopamine production in the substantia nigra of basal ganglia are basic pathophysiological changes that increase the activity of inhibitory nuclei, mainly including the activity of *γ*-aminobutyric acidergic neurons [15]. It was reported that *γ*-aminobutyric acid (GABA) in the corpus striatum of PD animals was increased with the decrease of dopamine [16]. It is well recognized that GABA is involved in the mechanism of general anesthesia. Recent evidence has demonstrated that propofol potentiates GABA (A) receptor on *β*3 homopentamers and *α*1*β*3 heteropentamers [17, 18], which might be one of the targets for general anesthesia. Muscimol, a GABA (A) receptor agonist, prolonged the duration of loss of the righting reflex and loss of the tail-pinch response after propofol administration in rats. Therefore, excessive activation of GABAergic neurons might enhance the anesthetic effect of propofol, thus reducing the propofol requirement for unconsciousness induction in PD patients.

Chronic medications in PD patients might also participate in the reduced propofol requirement for unconsciousness induction in PD patients. It was reported that levodopa, the mainstay of PD treatment, upregulated NMDA receptor subunit in several neuronal loci, which is one of the reasons for levodopa-induced dyskinesia [19, 20]. Knockout of NMDA receptor subunit in mice attenuated the hypnotic effect of propofol, indicating that NMDA receptor is an important target of propofol during anesthesia [21]. Therefore, levodopa administered to PD patients might also reduce the propofol requirement by increasing the density of NMDA receptor in the brain.

There are three main limitations in our study. The first one is that the sample size was relatively small, which might result in a false positive result. Secondly, we did not determine the real concentration of propofol in the patients' blood. The pharmacokinetic model of propofol used in the Marsh model might be not suitable for PD patients, and the predicted concentration of propofol might vary greatly from real concentration. Thus our conclusion must be based on the theory that PD patients shared the same propofol pharmacokinetic characters as the patients with intracranial tumor. Whether the pharmacokinetic model of propofol in PD patients is different from the others should be further investigated. Thirdly, patients with brain tumors were chosen as control, but not patients without PD undergoing DBS implantation for other reasons, because DBS has seldom been used in our hospital for other diseases. However, what we want to investigate is the propofol requirement for unconsciousness before surgery in PD and NPD patients. What kind of surgery would be performed should not be critical.

## 5. Conclusions

In conclusion, the present study demonstrates that the effective propofol concentration needed for induction of unconsciousness in 50% patients is reduced in PD patients. In other words, propofol requirement for induction of unconsciousness is reduced in PD patients. The mechanism remains to be explained by further studies.

## Figures and Tables

**Table 1:** Demographic data of the patients.

Parameters	PD group (*n* = 31)	NPD group (*n* = 31)	*P* value
Age (years)	57.4 ± 9.1	57.7 ± 8.5	0.99
Sex (male/female)	17/14	17/14	1.00
Body mass index (kg m^−2^)	22.2 ± 2.9	22.1 ± 4.1	0.97
ASA score (class II/III)	12/19	17/14	0.203
BIS	96.2 ± 2.5	95.2 ± 3.7	0.67
HR (bpm)	80.0 ± 8.7	77.2 ± 15.2	0.94
SBP (mmHg)	136.4 ± 17.6	132.8 ± 17.4	0.96
DBP (mmHg)	80.3 ± 12.1	78.3 ± 13.2	0.96

Values are presented as mean ± standard derivation or counts. Data were analyzed using paired Student's *t*-test or chi-square test. ASA: American Society of Anesthesiologists; BIS: bispectral index; HR: heart rate; SBP: systolic blood pressure; DBP: diastolic blood pressure.

**Table 2:** The anti-Parkinson medications taken by the patients with Parkinson's disease.

Medications	Number of uses (%)
Levodopa/benserazide	30 (96.8)
Trihexyphenidyl	10 (32.3)
Levodopa/carbidopa	6 (19.4)
Amantadine	6 (19.4)
Pramipexole	5 (16.1)
Entacapone	3 (9.7)
Bromocriptine	1 (3.2)
Rasagiline	1 (3.2)
